# Corticosterone Alters AMPAR Mobility and Facilitates Bidirectional Synaptic Plasticity

**DOI:** 10.1371/journal.pone.0004714

**Published:** 2009-03-05

**Authors:** Stéphane Martin, Jeremy M. Henley, David Holman, Ming Zhou, Olof Wiegert, Myrrhe van Spronsen, Marian Joëls, Casper C. Hoogenraad, Harmen J. Krugers

**Affiliations:** 1 MRC Centre for Synaptic Plasticity, Department of Anatomy, University of Bristol, Bristol, United Kingdom; 2 SILS-CNS, University of Amsterdam, Amsterdam, The Netherlands; 3 Department of Neuroscience, Erasmus Medical Center, Rotterdam, The Netherlands; L'université Pierre et Marie Curie, France

## Abstract

**Background:**

The stress hormone corticosterone has the ability both to enhance and suppress synaptic plasticity and learning and memory processes. However, until today there is very little known about the molecular mechanism that underlies the bidirectional effects of stress and corticosteroid hormones on synaptic efficacy and learning and memory processes. In this study we investigate the relationship between corticosterone and AMPA receptors which play a critical role in activity-dependent plasticity and hippocampal-dependent learning.

**Methodology/Principal Findings:**

Using immunocytochemistry and live cell imaging techniques we show that corticosterone selectively increases surface expression of the AMPAR subunit GluR2 in primary hippocampal cultures via a glucocorticoid receptor and protein synthesis dependent mechanism. In agreement, we report that corticosterone also dramatically increases the fraction of surface expressed GluR2 that undergo lateral diffusion. Furthermore, our data indicate that corticosterone facilitates NMDAR-invoked endocytosis of both synaptic and extra-synaptic GluR2 under conditions that weaken synaptic transmission.

**Conclusion/Significance:**

Our results reveal that corticosterone increases mobile GluR2 containing AMPARs. The enhanced lateral diffusion properties can both facilitate the recruitment of AMPARs but under appropriate conditions facilitate the loss of synaptic AMPARs (LTD). These actions may underlie both the facilitating and suppressive effects of corticosteroid hormones on synaptic plasticity and learning and memory and suggest that these hormones accentuate synaptic efficacy.

## Introduction

Individual neurons contain ∼10,000 synapses and the synapse-specificity of signalling and plasticity underlies the immense processing power of neuronal systems. The number and subunit compositions of synaptic AMPARs are stringently regulated because activity dependent changes in functional postsynaptic AMPARs contribute to the two main forms of synaptic plasticity that are believed to underlie learning and memory in the hippocampus [1], [2]. Long term potentiation (LTP) involves the activity-dependent recruitment of AMPARs to the postsynaptic membrane and a concurrent increase in AMPA-mediated transmission whereas long term depression (LTD) is a decrease in synaptic AMPAR function [3], [4].

The stress hormone corticosterone exert marked effects on learning and memory and both facilitating and impairing influences are described in the literature [5], [6]. Interestingly, corticosteroid hormones have profound effects on AMPAR function, synaptic transmission and plasticity via genomic and non-genomic pathways. Long-lasting effects are mediated via glucocorticoid receptors (GRs) which enhance AMPAR mediated miniature excitatory postsynaptic current (mEPSC) amplitude [7], impair NMDA receptor mediated long-term synaptic potentiation (LTP) [8] and facilitate long-term synaptic depression (LTD) [9], [10]. Rapid, non-genomic effects of corticosterone are mediated via high-affinity mineralocorticoid receptors (MRs), which act to enhance AMPAR mEPSC frequency [11] and facilitate synaptic potentiation [12].

Recently, using single particle tracking approaches it has been reported that corticosteroid receptor activation directly and long-lastingly impacts on AMPAR mobility. [13]. We investigated whether corticosterone alters the levels of synaptic AMPARs in basal conditions and under conditions that induce synaptic depression in hippocampal cultures. Under basal conditions, corticosterone increased GluR2 but not GluR1 containing AMPAR surface expression and enhanced mEPSPs. The increase was protein synthesis dependent and was accompanied by increased lateral diffusion. However corticosterone enhanced AMPAR endocytosis under conditions which promote LTD

## Materials and Methods

### Dispersed hippocampal neuronal cultures and immunocytochemistry

The experiments were carried out with permission of the local Animal Committee of the Erasmus Medical Center and University of Bristol. Primary hippocampal cultures were prepared from embryonic day 18 (E18) rat brains as described [14]. Cells were plated on coverslips coated with poly-D-lysine (30 µg/ml) and laminin (2 µg/ml) at a density of 75,000/well. Hippocampal cultures were grown in Neurobasal medium supplemented with B27, 0.5 mM glutamine, 12.5 µM glutamate and penicillin/streptomycin. At DIV13-20 hippocampal neurons were incubated with GluR1 (Calbiochem (1∶8) and GluR2 (Zymed (1∶80) N-terminal antibodies (10 µg/ml) at 37°C for 15 min [12]. After washing in DMEM medium, the neurons were fixed for 5 min with 4% formaldehyde/4% sucrose in phosphate-buffered saline (PBS). Neurons were then washed three times in PBS for 30 min at room temperature and incubated with secondary antibody conjugated to Alexa488 (1∶400) or Alexa568 (1∶400) in staining buffer without TritonX-100 (0.2% BSA, 0.8 M NaCl, 30 mM phosphate buffer, pH 7.4) overnight at 4°C. Neurons were then washed three times in PBS for 30 min at room temperature and mounted.

For total staining cells were fixed for 5 min with 4% formaldehyde/4% sucrose in phosphate-buffered saline (PBS). Next, cells were incubated with GluR1 (1∶5000) and GluR2 C-terminal antibodies (1∶500) [15] in staining-buffer with TritonX-100 overnight at 4°C. Neurons were then washed three times in PBS for 30 min at room temperature and incubated with secondary antibody conjugated to Alexa488 (1∶400) or Alexa568 (1∶400) in GDB with TritonX-100 for 2 h at room temperature and washed three times in PBS for 30 minutes.

Confocal images were obtained with sequential acquisition settings at the maximal resolution of the microscope (1024×1024 pixels). Morphometric analysis and quantification were performed using MetaMorph software (Universal Imaging Corporation). For details see supplementary materials and methods.

### Image analysis and quantification

Confocal images stained neurons were obtained with sequential acquisition settings at the maximal resolution of the microscope (1024×1024 pixels). Each image was a z-series of 6–10 images each averaged 2 times. The resulting z-stack was ‘flattened’ into a single image using maximum projection. The confocal settings were kept the same for all scans when fluorescence intensity was compared. Morphometric analysis and quantification were performed using MetaMorph software (Universal Imaging Corporation). For the quantification of surface antibody staining, images were acquired with use of a 40× objective with 1.0× electronic zoom and the average intensity of the soma and dendrites was measured in MetaMorph. Acquisition of the images as well as morphometric quantification was performed under “blinded” conditions. Statistical analysis was performed with student's t test assuming a two-tailed and unequal variation. N defined as the number of quantified neurons.

### Biotinylation assays

High-density hippocampal cultures were prepared as described previously (13After 14 DIV, neurons were treated with 100 nM CORT for 3 h or treated vehicle only and membrane and total fractions were prepared [16]. Immunoblotting was performed using a rabbit polyclonal antibody to GluR1 (Upstate; 0.6 µg/ml) and mouse monoclonal antibodies to GluR2 (Chemicon; 1 µg/ml), Transferrin receptor (Sigma 1 µg/ml), β-actin (Sigma; 0.5 µg/ml) and α-tubulin (Sigma; 0.5 µg/ml). Quantitative densitometric analysis was performed using NIH Image J.

### Electrophysiology

Coverslips were placed in a recording chamber mounted on an upright microscope (Nikon E600FN), continuously perfused with artificial cerebrospinal fluid (aCSF) (32°C, 2–3 ml/sec; containing in (mM): NaCl (120), KCl (3.5), MgSO_4_ (1.3), NaH_2_PO_4_ (1.25), CaCl_2_ (2.5), Glucose (10.0) and NaHCO_3_ (25.0), pH 7.4) and kept fully submerged. Whole cell patch clamp recordings were made using an AXOPATCH 200B amplifier (Axon Instruments, USA), with electrodes from borosilicate glass(1.5 mm outer diameter, Hilgerberg, Malsfeld, Germany). The electrodes were pulled on a Sutter (USA) micropipette puller. The pipette solution contained (in mM): 120 Cs methane sulfonate; CsCl (17.5); HEPES (10); BAPTA (5); Mg-ATP (2); Na-GTP (0.5); QX-314 (10); pH 7.4, adjusted with CsOH; pipette resistance was between 3–6 MΩ. Under visual control (40× objective and 10× ocular magnification) the electrode was directed towards a neuron with positive pressure. Once sealed on the cell membrane (resistance above 1 GΩ) the membrane patch under the electrode was ruptured by gentle suction and the cell was kept at a holding potential of −70 mV. The liquid junction potential caused a shift of no more than 10 mV, which was not compensated during mEPSCs recording. Recordings with an uncompensated series resistance of <15 MΩ and <2.5 times of the pipette resistance with a shift of <20% during the recording, were accepted for analysis. Data acquisition was performed with Pclamp 8.2 and analyzed off-line with Clampfit 9.0.

Miniature excitatory postsynaptic currents (mEPSCs) were recorded at a holding potential of −70 mV. Tetrodotoxin (0.25 µM, Latoxan, Rosans, France) and bicuculline methobromide (20 µM, Biomol) were added to the buffer to block action potential induced glutamate release and GABA_A_ receptor mediated miniature inhibitory postsynaptic currents (mIPSCs), respectively. During some recordings the non–NMDA-receptor blocker 6-cyano-7-nitroquinoxaline-2,3-dione (CNQX, 10 µM, Tocris) was perfused to confirm that the mEPSCs were indeed mediated by AMPARs. The events were identified as mEPSCs when the rise time was faster than the decay time. mEPSCs were recorded for 5 min in each cell.

### Sindbis virus preparation

Attenuated Sindbis virus expressing Super Ecliptic pHluorin (SEP)-tagged GluR2 (SEP-GluR2) [17], [18] was prepared and used as previously described [19]. Neurons were transduced at a MOI of 1 at 14–17 DIV and then returned to the incubator for an additional 24 h before use.

### Fluorescence imaging of SEP-GluR2 in living neurones

Protocols were as previously described [17], [18]. Briefly, live SEP-GluR2-expressing neurons (15–18 DIV) were preincubated for 3 hours in Neurobasal containing 30 nM corticosterone or vehicle and then transferred in Earle's buffer in the presence of either corticosterone (30 nM) or vehicle for live confocal imaging experiments. Neurons were placed on the heated stage (set at 37°C) of an inverted Zeiss Axiovert microscope and were continually perfused at 3 ml/min with warm Earle's±corticosterone solution. For low pH external solution, equimolar MES was used instead of HEPES and pH adjusted to 6.0. NH_4_Cl (50 mM) was used in place of equimolar NaCl to collapse pH gradient. Fluorescence was excited using 63× water-immersion objective (NA = 1.2) by 488 nm laser light and emission was detected through a 505 nm long pass filter. Time series were collected as repetitively scanned image stacks. Image stacks were then flattened using the maximum projection algorithm from the Zeiss LSM software. All SEP-GluR2 experiments include a brief (10 sec) low pH wash at the beginning to ensure that the fluorescence from the area of interest comes from surface-expressed AMPARs.

### Fluorescence recovery after photobleaching, FRAP

Fluorescence data were collected from regions of interest drawn around the fluorescence known to come from the cell surface of a spine (as shown by low pH wash). Data were individually normalized using a single-exponential fit to account for slow photobleaching of the whole image during the acquisition. FRAP was then expressed as a percentage of initial fluorescence (average of five images immediately before photobleaching) over time and fitted with the «Feder» equation that models Brownian diffusion in a membrane after photobleaching [18], [20]. The Mobile fraction values were extracted for each experiment from these fits (see also supplementary materials and methods).

### Synaptic and extrasynaptic AMPARs after NMDAR-dependent internalization

Brief bath application of 50 µM NMDA for 3 minutes at 37°C was applied to CORT-treated hippocampal cultured neurones to elicit a chemically induced form of long-term depression [21] and in dispersed hippocampal cultures, exposure to NMDA evokes internalization of synaptic GluR1- and GluR2-containing AMPARs [17], [21], [22], [23].

### Analysis of FRAP

Each fluorescence recovery after photobleaching (FRAP) dataset was expressed as a percentage of resting fluorescence (average of two to four images immediately before photobleaching) over time and fitted with the following equation that models Brownian diffusion in a membrane after photobleaching and incorporates the possibility of an immobile population [20]:
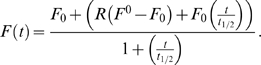

*F*_0_ is the fluorescence immediately after bleaching, *R* is the mobile fraction, *F* ^0^ is the fluorescence immediately before bleaching, and *t*_1/2_ is the half-time of the recovery curve. *R* was constrained in these fits to between 0 and 1.0. From these fits, the *R* and *t*_1/2_ values were extracted for each experiment. The *t*_1/2_ was used here as a comparative measure of the rate of diffusion since the regions that are being compared have the same or closely similar shape.

### Statistical analysis

Statistical analyses were calculated using Prism 4 (GraphPad software, Inc). Data are expressed as mean±s.e.m‥ Unpaired Student's t-tests and one-way ANOVA were performed with a Newman-Keuls post-test for multiple comparison data sets when required.

## Results

### GR activation increases AMPAR surface expression

**Figure 1:**
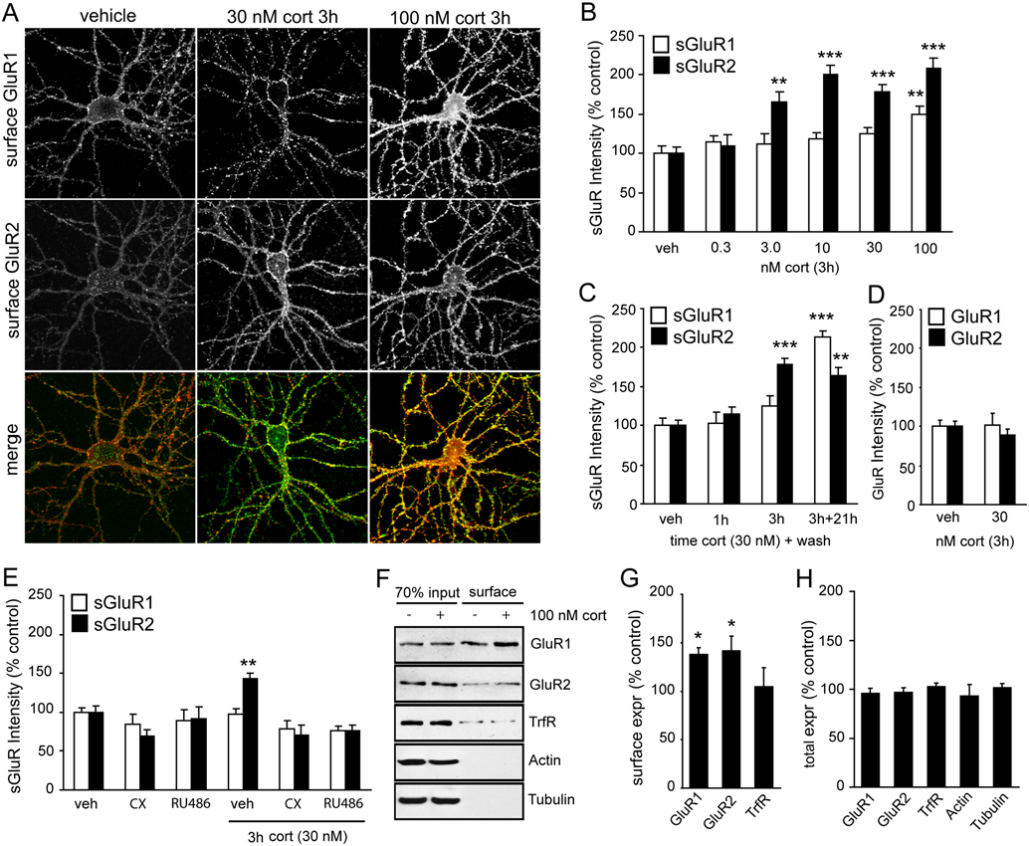
Glucocorticoid receptor activation promotes surface AMPA receptor expression. A) Representative images of hippocampal neurons at DIV13 treated with vehicle, 30 nM and 100 nM corticosterone for 3 h and stained for surface GluR1 (red) and GluR2 (green). B) Quantification of GluR1 and GluR2 intensity after treatment with vehicle and 0.3–100 nM corticosterone for 3 h. C) Quantification of surface GluR1 and GluR2 intensity after treatment with vehicle for 3 h and 30 nM corticosterone for 1 or 3 h. In addition cells were incubated for 3 h with CORT, washed and incubated in regular medium for 21 h (3 h+21 h). D) Quantification of total GluR1 and GluR2 intensity after treatment with vehicle and 30 nM corticosterone for 3 h. E) Quantification of surface GluR1 and GluR2 intensity of primary hippocampal neurons. Cells were treated with vehicle, 100 µM cycloheximide or 500 nM RU 38486 for 3 h followed 30 min later with vehicle or 30 nM corticosterone applications for 3 h. F) Representative Western blots show expression of GluR1, GluR2, transferrin receptor (TrfR), actin and tubulin in total and surface fraction of biotinylated primary hippocampal cultures treated with vehicle (−) or 100 nM corticosterone (+) for 3 h. G) Quantitative analysis of surface expression of GluR1, GluR2 and transferrin receptor (TrfR) in biotinylated primary hippocampal neurons treated with 100 nM corticosterone for 3 h. H) Quantitative analysis of total expression of GluR1, GluR2, transferrin receptor (TrfR), actin and tubulin in biotinylated primary hippocampal neurons treated with 100 nM corticosterone for 3 h.

Representative images of the effects of the treatment of primary hippocampal neurons with 30 and 100 nM corticosterone for 3 h on surface GluR1 and GluR2 immunolabelling is shown in Figure 1A. Quantitative dose response data (Figure 1B) indicate that both GluR1 and GluR2 surface expression are increased by corticosterone but that GluR2 is more sensitive and increases to a greater extent than GluR1. Significant increases in surface levels of GluR2 but not GluR1 were observed using 3 nM corticosterone, which is around the K_d_ value of the GR [24]. There were no detectable changes in surface expressed GluR1 or GluR2 after 1 h incubation with corticosterone (Figure 1C). Two hours after 1 hour incubation with corticosterone GluR2 levels were enhanced (data not shown), indicating that increased surface labelling is time dependent. The effects of corticosterone on AMPAR surface expression is also long lasting since surface GluR2 levels remain elevated 24 h after corticosterone application (and 21 h after washout). Interestingly, surface levels of GluR1 were also markedly increased at this time point (Figure 1C).

Total GluR2 (surface and intracellular pools) levels remained unchanged 3 h after incubation with 30 nM corticosterone (Figure 1D) indicating that corticosterone selectively enhance surface expression of AMPA receptors. This was confirmed using surface biotinylation in primary hippocampal cultures (Figure 1F–H).

**Figure 2:**
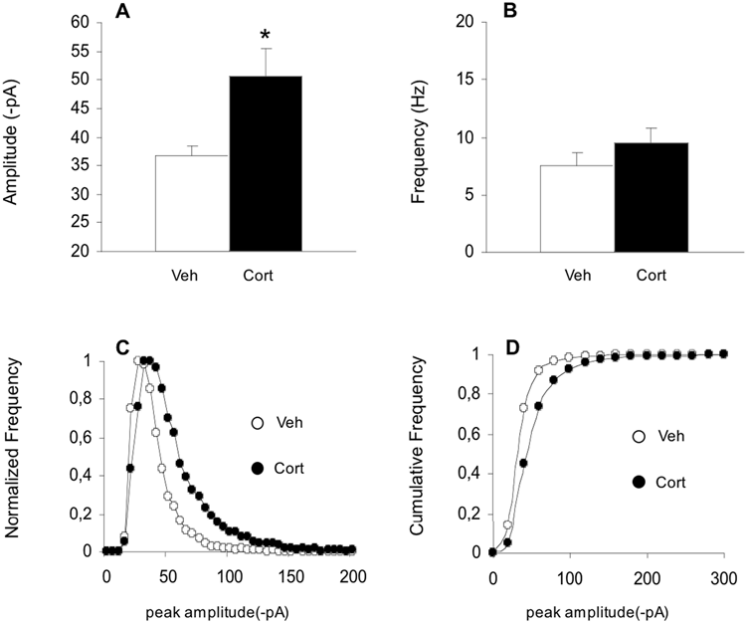
Corticosterone induces a delayed enhancement of the mEPSC amplitude. A) mEPSC amplitude and (B) frequency after treatment with vehicle or corticosterone. C) Normalized frequency histogram for the distribution of the amplitude of mEPSCs in hippocampal primary neurons after control treatment or treatment with corticosterone. A shift toward larger amplitudes was observed after hormone treatment. D) Cumulative frequency histogram shows a marked shift toward larger amplitude mEPSCs after corticosterone treatment. ^*^P<0.05.

The slow and persistent nature of the changes in AMPAR surface expression is suggestive of a gene-mediated pathway involving nuclear receptors. Consistent with this, application of the GR antagonist RU38486 (500 nM) completely blocked the corticosterone-induced increase in GluR2 surface expression and co-treatment of corticosterone with the protein synthesis inhibitor cycloheximide (100 µM) also fully prevented the effects of corticosterone on GluR2 surface expression (Figure 1H). Electrophysiological recordings demonstrate that corticosterone-treated cells displayed increased mEPSC amplitude (50.5 pA±4.9 for corticosterone vs. 36.8 pA±1.7 for control, Figure 2A) whereas the frequency in dispersed hippocampal cultures remained unchanged upon corticosterone treatment (Figure 2B). Frequency histograms revealing enhanced mEPSC amplitude after corticosterone treatment are shown in Figure 2C & D. Importantly, this indicates that corticosterone induced increments in surface GluR2 labelling are accompanied by enhanced AMPAR mediated synaptic transmission.

### Corticosterone increases surface mobility of GluR2

**Figure 3:**
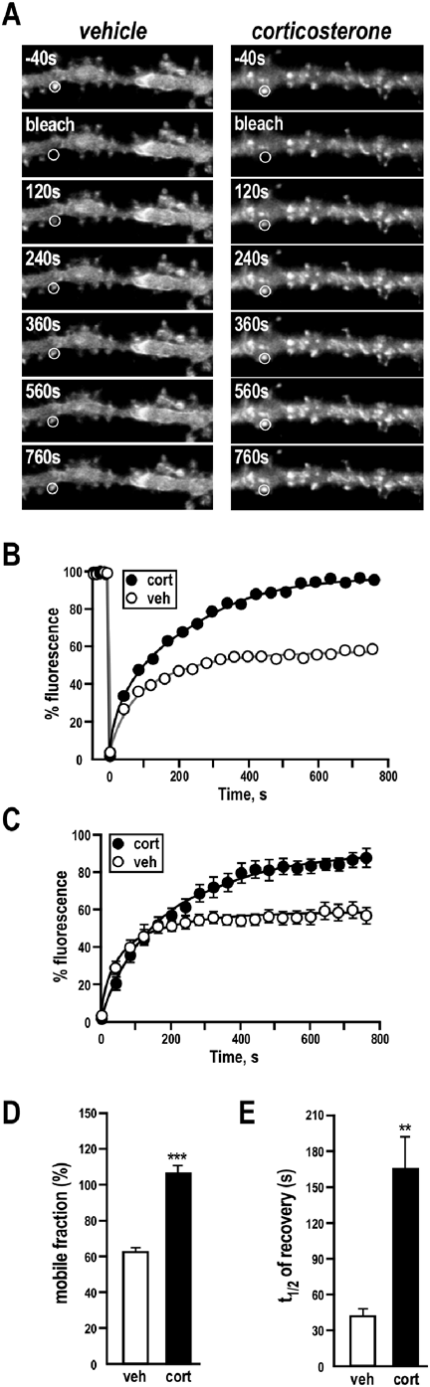
FRAP of SEP–GluR2 in hippocampal neurons shows that corticosterone treatment alters AMPAR lateral diffusion. A) Images from experimental time course showing that photobleached AMPARs on the surface of spine heads (in white circle) are exchanged for fluorescent SEP-GluR2. B) Normalised FRAP curves from experiments showed in A indicating that corticosterone treatment (30 nM) induces fully recovery of fluorescence after photobleaching over the course of 13 min as bleached AMPARs are completely exchanged with unbleached AMPARs. C) Normalised pooled and averaged FRAP curves from 10 spines from vehicle and corticosterone (30 nM) treated hippocampal neurones. D) Histogram showing mean±SEM of SEP–GluR2 mobile fractions in spine heads under vehicle and corticosterone conditions. Note that SEP-GluR2 FRAP corresponding to AMPAR exchange in spines is greatly enhanced by pre-incubation with 30 nM corticosterone. *** p<0.0001 vs. control. E) Histogram showing mean±SEM of half time of SEP–GluR2 fluorescence recovery in spine heads under vehicle and corticosterone conditions. ** p<0.001 vs. control.

One explanation for the increase in surface GluR2 puncta and mEPSCs is that corticosterone recruits extrasynaptic surface expressed AMPARs to synapses. We therefore performed fluorescence recovery after photobleach (FRAP) experiments using SEP–GluR2 [18] to visualise the effects of corticosterone on surface motility in spines. As expected spine heads contained bright SEP-GluR2 fluorescence under resting conditions (Figure 3A top panel). The extent of and time course of fluorescence recovery following bleaching at individual spine heads of similar shape provides information of the fraction of SEP-GluR2 that is mobile and the residence time of synaptic AMPARs [25]. There was a partial recovery of fluorescence in bleached spines due to the exchange of bleached surface SEP-GluR2 with unbleached SEP-GluR2 from the dendritic shaft (Figure 3) via lateral diffusion [18].

In control cells FRAP plateaus to ∼60% (61.8%±2.5) of pre-bleach levels within 3–4 min. Corticosterone treatment (30 nM) caused a full recovery of fluorescence after photobleaching (107.7%±4.2) over the time course of our experiments (Figure 3B–D). Taken together, these data suggest a complete loss of the immobile pool of synaptic AMPA receptor upon corticosterone treatment (i.e. every single SEP-GluR2 subunit is free to move inside/outside of the spine in corticosterone treated neurons. Furthermore, the half time of fluorescence recovery in spines (Figure 3E) was ∼4 times higher for corticosterone-treated cells (166.6 s±26.9) than for control cells (40.5 s±8.2) indicating that corticosterone treatment acts to remove the immobile fraction of synaptic AMPARs and that it takes longer in presence of corticosterone to fully exchange the synaptic population of SEP-GluR2.

### Effects of corticosterone on AMPAR trafficking during LTD

**Figure 4:**
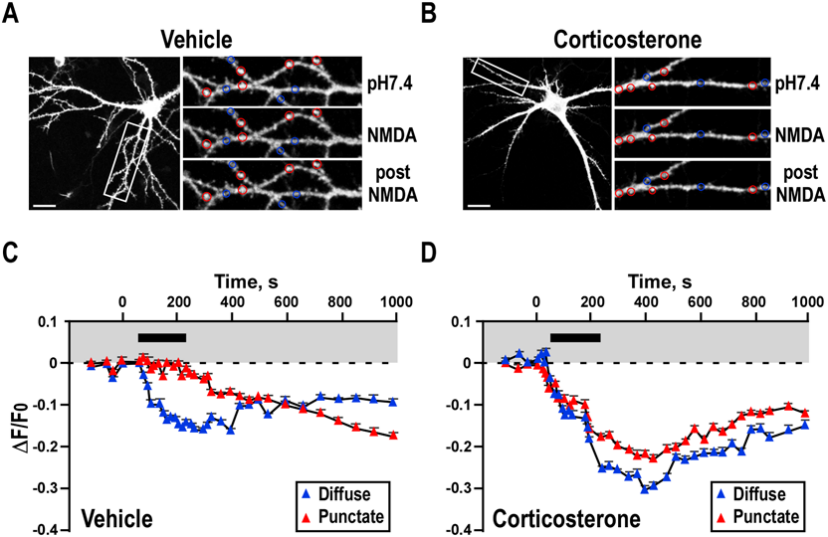
Corticosterone treatment dramatically modifies the endocytic properties of AMPARs. A) Rapid endocytosis of synaptic (punctate) and extrasynaptic (diffuse) AMPARs induced by activation of NMDARs in control hippocampal neurons. Scale bar, 20 µm. B) Rapid endocytosis of synaptic (punctate) and extrasynaptic (diffuse) AMPARs induced by activation of NMDARs in corticosterone-treated hippocampal neurons. Scale bar, 20 µm. C) Binned and averaged fluorescence values from punctate (red) and diffuse (blue) regions during and after NMDAR stimulation in control treated cells. Data reflect error bars show±SEM (4 cells for each condition with 14 punctate and 17 diffuse regions). D) Binned and averaged fluorescence values from punctate (red) and diffuse (blue) regions during and after NMDAR stimulation in corticosterone treated cells. Data reflect error bars show±SEM (4 cells for each condition with 14 punctate and 17 diffuse regions).

A recent study using single particle tracking approaches reported that short corticosterone application (10–20 min) increases GluR2-AMPAR surface mobility in a time-dependent manner and increased residency of GluR2 at synapses, which occludes increases in GluR2 synaptic content induced by a chemical LTP protocol [13]. LTD is the converse process to synaptic strengthening and is also mediated by synaptic AMPARs. We therefore investigated the effects of corticosterone on NMDAR-induced LTD in hippocampal cultured neurons [21]–[23]. As we have reported previously [14] there was a marked difference in the behavior of punctate synaptic and diffuse extra synaptic SEP–GluR2 during NMDA application (Figure 4). In control cells fluorescence from punctate, synaptic regions was relatively stable during the 3 min period of NMDA application but then slowly declined after NMDA removal. In stark contrast, non-punctate extrasynaptic SEP–GluR2 fluorescence decreased immediately and rapidly as soon as NMDA was applied consistent with rapid internalization of AMPARs from the extrasynaptic membrane into an acidic intracellular compartment [17]. After NMDA washout, the fluorescence immediately began to recover toward pre-stimulation levels.

Pretreatment with corticosterone had dramatic effects on AMPAR trafficking in LTD (Figure 4). In corticosterone-treated cells the punctate synaptic SEP-GluR2 fluorescence started to decrease immediately on exposure to NMDA with a time course similar to the decrease in diffuse non-synaptic SEP-GluR2 (Figure 4B, D). Further, there was a much greater initial loss of SEP-GluR2 fluorescence from non-punctate regions. In direct contrast to control cells, the punctate and diffuse SEP-GluR2 in corticosterone-treated cells showed similar loss of SEP-GluR2 fluorescence kinetics upon NMDAR activation. These results are consistent with corticosterone both increasing and recruiting the normally synaptic immobile fraction into the freely diffusing pool of receptors (Figure 3). Further, these data imply that corticosterone facilitates NMDAR-dependent AMPAR endocytosis within the spine and allows rapid exchange of surface synaptic expressed receptors from spines to endocytic sites on the dendritic shaft.

## Discussion

It has recently emerged that corticosterone treatment directly and long-lastingly impacts on AMPAR lateral mobility [13]. Here we report that prolonged corticosterone treatment increases GluR2 surface expression in a time and concentration dependent manner. We observed no change in GluR2 after 1 h of corticosterone but pronounced effects after 3 h suggestive of a mechanism involving GR-mediated transcriptional regulation. Consistent with this, corticosterone had no effect on GluR2 in the presence of a protein synthesis inhibitor. Additionally, using electrophysiological approaches, we found that the increased levels of surface GluR2 in corticosterone-treated cells result in increased functional synaptic AMPARs.

**Figure 5:**
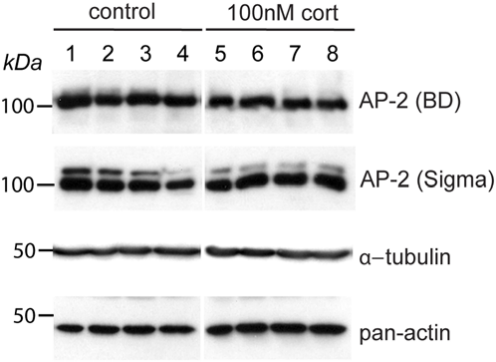
AP-2 levels in primary hippocampal neurons treated with corticosterone. Hippocampal neurons (DIV 22) were treated with vehicle (lane 1–4) or 100 nM corticosterone (lane 5–8) for 3 h and harvested directly in sample buffer. The immunoblots were probed with two different anti-AP-2 (from Sigma and BD biosciences), anti-α-tubulin and anti-pan-actin antibodies. The positions of molecular weight standards (kDa) are indicated at left. No difference in AP-2 expression levels between control and corticosterone treated neurons is observed.

Despite the marked changes in surface levels of GluR2 total levels were unchanged. These results could be interpreted to suggest that newly synthesized GluR2 is rapidly targeted to the cell surface and there is a corresponding increase in degradation of intracellular GluR2 to maintain balance. A more likely alternative explanation, however, is that GR activation does not directly target the GluR2 encoding gene. Indeed, in preliminary studies no transcriptional regulation of any of the four AMPAR subunits (GluR1-4) was observed with qPCR in tissue prepared from hippocampal slices 3 h after corticosterone treatment (unpublished observations). Rather we envisage a system in which GR activation alters genes encoding proteins involved in regulating GluR2/3 delivery and/or membrane anchoring. There are multiple potential candidates including TARPS, PICK1, GRIP or NSF and AP2 [21], [26]–[29]. Interestingly, AP2 is one of the down-regulated genes picked up in a microarray survey using corticosterone-treated hippocampal slices of rats [30]. However, we could not observe a significant difference in total AP2 protein levels in 100 nM glucocorticoid treated hippocampal cultures compared to controls (Figure 5).

The corticosterone mediated enhancement of surface GluR2 levels can be attributed to increased exocytosis and/or reduced endocytosis of AMPARs. In addition, using fluorescence imaging of SEP-GluR2 in living neurons we noted that corticosterone mobilizes normally synaptically anchored surface expressed AMPARs. One advantage of live cell imaging of SEP-GluR2 over single particle tracking methods is that it samples populations of receptors and allows simultaneous analysis of both mobile and immobile populations [25]. We demonstrate an apparent complete loss of the immobile pool of synaptic AMPA receptor upon corticosterone treatment (i.e. every SEP-GluR2 subunit is free to move in corticosterone treated neurons) and that it takes under these conditions longer to exchange half of the bleached SEP-GluR2 population (which is double in corticosterone-treated cells compared to control neurons). The absence of a detectable immobile fraction of SEP-GluR2 following corticosterone treatment suggests that corticosterone effectively removes the diffusion barrier that normally limits AMPAR entry and egress between the spine and shaft membrane compartments.

The physiological significance of the profound effects corticosterone has on GluR2 lateral mobility is likely to be widespread and dependent on the cellular and synaptic context. Under basal conditions the corticosterone-invoked increase in freely mobile GluR2 facilitates recruitment and leads to increased synaptic efficacy. Furthermore, the extracellular N-terminal domain of GluR2 can interact directly with the cell adhesion molecule N-cadherin to promote the formation and growth of dendritic spines [31], [32]. Via these pathways corticosterone could potentially facilitate learning and memory processes when corticosterone levels are elevated in the context of the learning event [5], [33]. In addition, enhanced AMPAR levels 2–3 hours after corticosterone may occlude LTP [5], [10], [34] and hinder learning processes *after* exposure to stress [5].

Further, our data also indicate that corticosterone facilitates NMDAR-invoked endocytosis of both synaptic and extra-synaptic SEP-GluR2. Thus, under these circumstances GR activation will reduce synaptic AMPAR content leading to decreased synaptic efficiency [10].

Thus, depending on the synaptic context corticosterone can facilitate and reduce synaptic efficacy. This suggests that in stressed situations corticosterone can facilitate normal AMPAR trafficking processes that may accentuate learning and memory processes.
